# How Effective Are Non-Frictional Techniques Compared to Sliding Techniques in the Retraction of Upper Anterior Teeth When Using Buccal Fixed-Appliance Therapy? A Systematic Review

**DOI:** 10.3390/jcm12216757

**Published:** 2023-10-25

**Authors:** Mohammad Naem Kheshfeh, Mohammad Younis Hajeer, Mhd. Firas Al Hinnawi, Mohammed Adel Awawdeh, Ali S. Aljhani, Nora Alhazmi

**Affiliations:** 1 Department of Orthodontics, Faculty of Dentistry, University of Damascus, Damascus P.O. Box 16046, Syria; m.khushfa10@gmail.com; 2 Biomedical Engineering Department, Faculty of Electrical and Mechanical Engineering, University of Damascus, Damascus P.O. Box 16046, Syria; firas.alhinnawi@damascusuniversity.edu; 3 Preventive Dental Science Department, College of Dentistry, King Saud bin Abdulaziz University for Health Sciences (KSAU-HS), Riyadh 11426, Saudi Arabia; jhania@mngha.med.sa (A.S.A.); nora.alhazmi2012@gmail.com (N.A.); 4 King Abdullah International Medical Research Center, Ministry of National Guard Health Affairs, Riyadh 11481, Saudi Arabia; 5 Dental Services King Abdulaziz Medical City, Ministry of National Guard Health Affairs, Riyadh 11426, Saudi Arabia; 6 College of Medicine & Dentistry, Ulster University, Birmingham B4 6BN, UK

**Keywords:** non-frictional technique, sliding technique, adult, anterior teeth retraction

## Abstract

Methods for retracting the anterior teeth are divided into frictional methods and non-frictional methods. However, evidence regarding the superiority of one technique over the other is still lacking in the available literature. Therefore, we aimed to evaluate the current evidence regarding the effectiveness of frictional methods of anterior teeth retraction compared to the non-frictional ones. The extracted data included the mechanism of application of the retraction force and its intensity, the observation period, follow-up records, and outcome measures. Ten studies were included in this review; the results did not favor a specific technique regarding the rate of orthodontic tooth movement and loss of anchorage during canine retraction, although a preference was shown for the sliding technique in the rate of en-masse retraction (0.74 versus 0.39 mm/month) and the anchorage control during the retraction of the incisors (0.5 versus 0.1 mm/month). The control of the incisor’s torque during the en-masse retraction was higher when frictionless techniques were used (−12° versus −7°). Regarding the rate of orthodontic tooth movement, the non-frictional technique is characterized by a high sensitivity to the quality of the design, and the sliding technique was generally effective. As for controlling the torque of the incisors, the preference is for the non-frictional technique. Overall, there is a need to conduct more studies with an appropriate design.

## 1. Introduction

Cases involving the retraction of the anterior teeth constitute a large proportion of the cases that visit the orthodontist in daily practice [1]. Camouflage therapy is a treatment option followed in most cases in adults, and this is performed by extracting the first premolars to relieve the disturbance in the anteroposterior direction. Extraction of the first premolars is followed by a retraction, which can either be in a single phase, called an en-masse retraction [2], or in two phases, in which the canines are retracted individually before the incisors are retracted [3]. The primary goal during the retraction stage is to achieve anterior teeth retraction with the best possible control of the tooth position and the shortest time for correction [4].

The retraction of anterior teeth can be accomplished with several methods when using buccal (vestibular) fixed-appliance therapy. In the two-step retraction technique, the canine is first moved backward using a loop-based sectional archwire originating from the first molars on each side of the dental arch [5], or by pulling the canine backward along a solid archwire in a sliding motion, employing a power chain or a closed coil spring [6]. In the second step, incisors can be retracted using a continuous archwire supplemented with T-shaped or inverted-L-shaped loops [7], a utility arch [8], or by employing the sliding mechanism along a solid archwire [9]. On the other hand, the en-masse retraction of the upper six teeth can be accomplished in two different ways. The first way is based on using continuous archwires provided with loops (of any kind) that can be activated to move the whole block of teeth backward [7], whereas the second way is dependent on the sliding mechanism on continuous archwire, which is always associated with friction during the retraction of the six teeth backward [3].

The technique that does not depend on the loops (the sliding mechanism) differs from the one that depends on the loops in the presence of friction. The friction when the tooth is moved is generated by several factors such as the properties of the contact surface between the archwire and the bracket slot, the shape of the archwire cross-section, type, and the force of ligation [10,11,12,13]. In sliding mechanics, the movement can be hindered if the friction is high, such as if the clearance between the archwire and the slot of the bracket/tube is small [14], or if the archwire forms a large angle with the slot of the bracket/tube [15]. On the other hand, the term ‘frictionless movement’ includes techniques that use loops acting as springs to move one tooth (e.g., canine) or a group of teeth (e.g., four upper or lower incisors). The orthodontic literature is full of examples such as Burstone’s T-loop, Ricketts’s spring, or Gjessing’s spring [5,16,17].

In canine retraction, some researchers claim the advantages of moving the teeth using sectional archwires with embedded loops in controlling the 3D spatial canine position. The use of sectional archwires is claimed to help avoid the deepening of the bite, eliminate friction problems, and accelerate movement [17,18]. Many proponents of the sliding technique claim that it is easier, faster, does not require a large clinical time, is simpler, and has fewer complications [19].

In the en-masse retraction of anterior teeth, some clinical reports have indicated that the retraction using segmented techniques secures better control of the movement of the teeth and is more predictable if the work is performed with great accuracy, as working with it requires full knowledge and high control of the force systems generated by it [20]. The location of the T-loop on the arch contributes greatly to the determination of the force system resulting from the activation of the spring [20]. Whereas in the sliding technique, it is practically difficult to know the system of force applied due to friction [21], as it causes rapid changes in the location, direction, and intensity of the stress generated within the periodontal ligament due to friction [22]. Additionally, because the distance between the canine bracket and the second premolar bracket is limited, achieving a differential closure of the distance is also difficult, so most doctors use the sliding technique here if the anchorage required is B, as it may require an enhanced anchorage for group A by applying additional anchorage items [18].

Studies comparing frictionless methods with sliding ones have shown some contradictory results, as one study found that the retraction with the loop-based technique via the Gjessing retraction spring takes less time than the sliding technique through the elastic chain in canine retraction cases [17]. In another study, it was found that the sliding technique on a continuous archwire using a coil spring ensures a faster dental movement than the retraction using the loop-based technique through T-loops [23].

After excavating the orthodontic literature, no published systematic review was found that compared sliding versus loop-based techniques, and since there is no previous systematic review that has examined this topic and these comparisons, it was desirable to conduct this review to answer the following focused review question: “Which is more effective in the retraction of anterior teeth: friction-based sliding mechanisms or loop-based ones?”

## 2. Materials and Methods

The Preferred Reporting Items for Systematic Reviews (PRISMA) guidelines were followed when writing up this report. This systematic review was registered in the PROSPERO database (registration number CRD42023452259) on 17 August 2023.

### 2.1. Eligibility Criteria

Eligibility criteria were established based on the PICOS framework. The target population was patients with any malocclusion that required the extraction of the first premolars followed by the retraction of the anterior teeth. The intervention was any type of sectional non-frictional technique for the retraction of canines, incisors, or the six anterior teeth. The comparison was any type of sliding retraction technique. The outcomes of interest were orthodontic tooth movement rate, control of angulation (tipping), inclination (torque), root resorption, and anchorage loss. All included studies were clinical studies of either split-mouth or parallel-group clinical studies, published exclusively in English.

### 2.2. Search Strategy

The electronic literature review was carried out utilizing the following databases, PubMed^®^, Scopus^®^, EMBASE^®^, the Cochrane Central Register of Controlled Trials, Web of Science™, and Google™ Scholar, for all studies published up to 28 February 2023. The keywords that were used in the electronic search are given in Table 1, whereas the details of the search strategy are presented in Table A1 (Appendix A).

### 2.3. Study Selection and Data Extraction

The two review authors (M.N.K. and M.Y.H.) extracted studies according to the inclusion criteria; when there was a conflict of opinion, the third author (M.A.A.) was asked to resolve the matter until an agreement was reached. The authors of the retrieved articles were contacted when there were inquiries and to obtain additional clarifications. Initially, all articles were entered based on title and abstract. In the next step, the full text of all articles selected for examination was reviewed. Articles that did not meet at least one of the eligibility criteria were excluded from the review. Finally, the articles included were determined by the predefined criteria. From all articles, the following information was extracted: names of authors, study design, sample size, the mean age of patients, the mechanism of application of the retraction force and its intensity, observation period, follow-up records, and outcome measures.

### 2.4. Assessment of Risk of Bias in Individual Studies

The quality of the articles was evaluated by the two authors (M.N.K. and M.Y.H.). When there was a disagreement, the third author (M.A.A.) was consulted to reach an agreement. The Cochrane’s Risk of Bias tool was used by the authors to judge (high, low, or unclear) the risk of bias from five domains (selection, performance, attrition, reporting, and other) for individual items in randomized controlled trials (RCTs) [24]. The overall risk of bias was determined for individual studies. The risk of bias was considered low when all fields indicated a low risk of bias and considered unclear or high when one or more fields indicated an unclear or high risk of bias, respectively. The ROBINS-I tool was used for non-randomized trials to judge (low, moderate, serious, critical, the risk of bias or no information) the risk of bias from seven domains (bias due to confounding, bias in selection of participants into the study, bias in classification of interventions, bias due to deviations from intended interventions, bias due to missing data, bias in measurement of outcomes, and bias in selection of the reported result) [25]. The overall risk of bias was also determined for individual studies.

## 3. Results

### 3.1. Literature Search Flow and the Retrieved Studies

**Figure 1:**
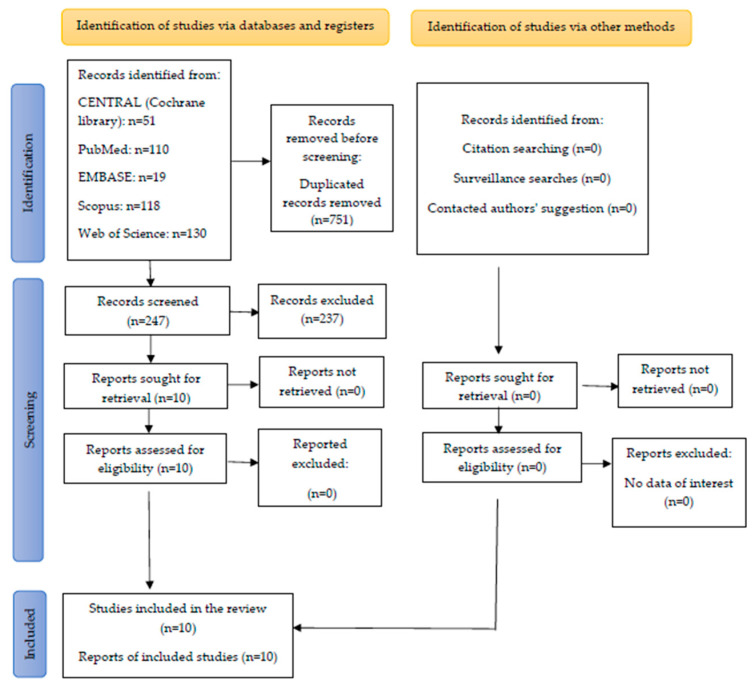
PRISMA flow diagram of study identification, screening, and inclusion.

Nine hundred and ninety-eight studies were found through an electronic search. Two hundred and forty-seven articles were carefully checked after removing duplicates. The titles, abstracts, and full texts of the articles were screened to search for articles that met the inclusion criteria. All articles that did not meet these criteria were excluded. Finally, the systematic review included ten articles [16,17,23,26,27,28,29,30,31,32]. The PRISMA flow diagram of study identification, screening, and inclusion is given in Figure 1.

### 3.2. Characteristics of the Included Studies

The characteristics of the ten included studies are presented in Table 2. Four of the studies were randomized controlled clinical trials [7,27,31,32], one was a non-randomized split-mouth study [17,23], and five were non-randomized two-group comparative studies [16,26,28,29,30]. The studies included 255 adult patients. Seven studies (70%) reported gender distribution within the sample; the male to female ratios were varied, being approximately 1:2 in two studies [23,29], 1:3 in two studies [17,31], and three articles included almost all female patients [27,30,32]. Three studies (30%) did not report gender distribution for the included patients [16,26,28]. Five studies (50%) mentioned the ages of the samples included in the study, with a mean age range from 15 to 25 years in the recruited samples [17,27,29,30,32].

Five studies included Class I malocclusion cases with bimaxillary dentoalveolar protrusion that required the extraction of first four premolars [16,26,27,30,32]; four studies included Class II malocclusion cases that required the extraction of the upper first premolars only for retracting the upper anterior teeth [17,23,28,29]; one study included both types of malocclusions [31].

Four out of ten comparative studies evaluated the retraction of the upper canines. Two studies examined the retraction of the upper incisors [27,32], and four comparative studies evaluated the retraction of the upper six anterior teeth together (en-masse retraction) [26,28,30,31].

Four studies investigated the retraction of the canines; two of these studies (50% of the canine retraction studies) were of a split-mouth design [16,23], and two studies (50% of the canine retraction studies) had a parallel-group design [17,28]. Two studies investigated incisor retraction and were RCT studies [27,32]. Four studies of them investigated en-masse retraction; one was an RCT [31], one was a comparative study [28], and two were retrospective studies [26,30].

Bracket prescriptions differed between studies. Two out of ten studies used the standard Edgewise brackets with a slot height of 0.018 inches [17,30]. Seven of the studies used brackets with a slot height of 0.022 inches, two of them used the Roth prescription [27,32], two of them used the MBT prescription [23,31], and three studies did not mention the bracket prescription [16,26,28]. One of ten studies used a combination of the two types [30].

The diameters of the stainless steel archwires used to retract the canines by sliding mechanics were 0.018-inch in 0.018-inch brackets in one study [16] and 0.022-inch brackets in another study [29], rectangular 0.018 × 0.025-inch in 0.022-inch brackets, or 0.016 × 0.022-inch in 0.022-inch brackets. The diameter of the used stainless steel archwire for incisor retraction by sliding mechanics was 0.017 × 0.025-inch in 0.022-inch brackets [27,32]. The diameters of the used stainless steel archwires for en-masse retraction by sliding mechanics were 0.016 × 0.022-inch in 0.018-inch brackets [30], or 0.019 × 0.025-inch in 0.022-inch brackets [26,28,31].

The designs of the springs used to retract the canines varied; a Gjessing’s retraction spring was used and its effectiveness was compared to sliding on the 0.018-inch S.S wire [17]. The retraction efficacy was compared using T-loop springs to sliding both on 0.018 × 0.025-inch [18] and on 0.016 × 0.022-inch stainless steel [23]. The retraction using a Ricketts’s canine retraction spring versus sliding on a 0.018-inch archwire was also compared [29]. The design of the spring used for incisor retraction via a frictionless technique was a T-loop fabricated with 0.017 × 0.025-inch TMA, and its effectiveness was compared to sliding on 0.017 × 0.025-inch stainless steel [27,32]. The design of the spring used for en-masse retraction via the frictionless technique was a T-loop or mushroom-looped continuous archwire fabricated with 0.017 × 0.025-inch TMA, and its effectiveness was compared with the sliding method on a straight 0.019 × 0.025-inch stainless steel [26,28] or with a mild curve of Spee [31], respectively.

Three studies (30% of all included studies) evaluated the canine retraction rate [17,23,29]; two studies (20% of all included studies) investigated anchorage loss during canine retraction [16,17]; two studies (20% of all included studies) investigated the change in the canine tip (angulation) and rotational movements during retraction [17,29]; two studies evaluated the anchorage loss following incisor retraction [27,32]; one study evaluated the rate of incisor retraction [32]; and one study evaluated the torque changes of the incisors, ANB°, and B° change after incisor retraction [27]. Two studies evaluated the rate of en-masse retraction [28,31]; four studies (40% of all included studies) evaluated the molar anchorage loss of en-masse retraction [26,28,30,31]; and three studies (30% of all included studies) evaluated the torque changes of en-masse retraction [28,30,31].

Many assessment tools are used to study the variables, and two studies used more than one measurement tool [28,31]. Five studies (50% of all included studies) used dental casts to study some of the variables after they were scanned or photographed and inserted into software for analysis [17,28,29,31,32]. These variables included the rate and anchorage loss of en-masse retraction [28,31] and the rate of retraction and anchorage loss of incisor retraction [32]. Also, they included the rate and amount of canine retraction [17,29], rotation [17,29], tipping [17,29], and anchorage loss [17].

Five studies (50%) of all included studies used lateral cephalometrics to study some of the variables; these variables included molar anchorage loss [16,26,30,31], and SNA°, SNB°, ANB°, SN-MP°, SN-U1°, SN-L1°, U1 and U6 position, L1 and L6 position, overjet, and overbite changes after the en-masse retraction [28,30,31].

Two studies (20% of all included studies) used cone-beam computed tomography (CBCT) to study variables such as anchorage loss, torque changes of the incisors, ANB°, and B° change after incisor retraction [27], and to evaluate the amount of canine retraction [23], canine tipping, canine rotation, and the root resorption of canines [23].

### 3.3. Risk of Bias of Included Studies

**Figure 2:**
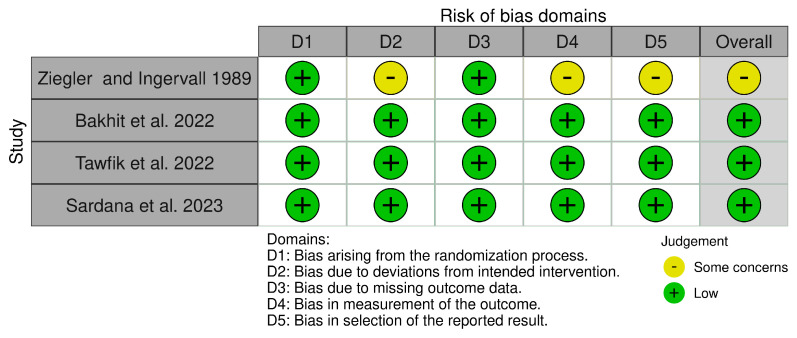
Risk of bias of the included randomized controlled trials [17,27,31,32].

**Figure 3:**
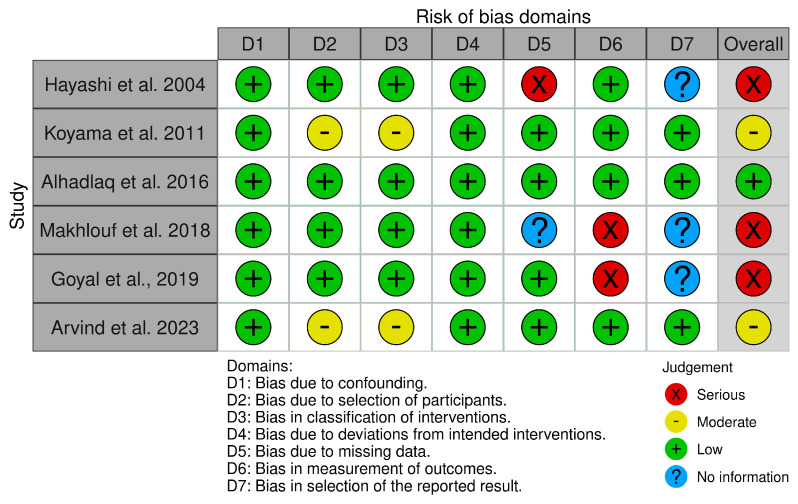
Risk of bias of the included non-randomized comparative studies [16,23,26,28,29,30].

Of the randomized trials, three trials were classified as low risk of bias [27,31,32], and one trial was classified as having some concern due to insufficient information to counter the selectivity of the reported results [17]. Of the non-randomized trials, one study was classified as low risk of bias [16], two trials were classified as having a moderate risk of bias due to some aspects of intervention status designations being determined retrospectively [26,30], and three trials were classified as having a serious risk of bias [23,28,29]. Figure 2 and Figure 3 summarize the overall risk of bias in the included studies, whereas the reasons behind each judgment are given in Tables S1 and S2.

### 3.4. Effects of Intervention

The findings of the retrieved studies are qualitatively synthesized under three categories: canine retraction, incisor retraction, and en-masse retraction of the six upper anterior teeth. The collected findings are summarized in Table 3.

#### 3.4.1. First: Canine Retraction

##### Rate of Canine Retraction

Three studies compared the retraction rate of canines between the two techniques [17,23,29]. The results varied between these studies. In two studies, the canine retraction was more rapid in the loop-based techniques at a rate of 1.91 and 1.97 mm/month compared to 1.4 and 1.81 mm/month in the continuous techniques when the Gjessing’s retraction spring and Ricketts’s canine retraction spring were used as the loop method, respectively [17,29]. In the third study, the retraction via the sliding technique was faster than the loop-based technique at a rate of 0.7 against 0.1 mm/month [23]. It was not possible to perform a meta-analysis due to the different tools used for the retraction.

##### Canine Tipping and Rotation Change during Canine Retraction

Two studies investigated the change in the canine tipping angle and its rotation after retraction. These two studies found that the tipping change was insignificantly greater in the sliding group, while the amount of rotation that occurred was greater in the loop-based retraction group [17,29]. Ziegler and Ingervall found that the mean tipping change when using a Gjessing spring was significantly less compared to when sliding on 0.018 stainless steel archwire (a mean of 0.7°/mm versus 1.4°/mm, respectively) [17]. As for the rotation, there were no essential differences between the two techniques [17]. Hayashi et al. found that Rickett’s maxillary canine retractor caused an apparent mean rotation of about 22 degrees compared to 4 degrees when sliding mechanics on 0.018-inch stainless steel archwires were used in the opposing group [29].

##### Anchorage Loss following Canine Retraction

Two studies investigated anchorage loss after retraction [16,17]. One of the two studies concluded that anchorage loss when using the Gjessing spring was similar to that when using the sliding technique on 0.018-inch stainless steel, with a mean of 0.09 mm per 1 mm of canine retraction versus 0.07 mm per 1 mm of canine retraction, respectively [17]. The other study showed that when using the sliding technique on 0.018 × 0.025-inch stainless steel, there was an average anchorage loss of 4.5 mm [16]. When using a T-loop fabricated from 0.019 × 0.025-inch TMA (RMO) with 0.022 × 0.025-inch brackets, a distal movement of the first molar was obtained with an average of 0.7 mm [16].

##### Root Resorption following Canine Retraction

A single study investigated the outcome between the two techniques [23]. The rate of canine root resorption upon retraction by a 0.017 × 0.025-inch TMA T-loop spring was insignificantly greater than sliding on a 0.016 × 0.022-inch stainless steel archwire (a mean of 0.275 mm/month with a total amount of 1.1 mm versus 0.05 mm/month with a total amount of 0.2 mm, respectively) [23]. Resorption was investigated by measuring the change in canine length using CBCT images [23].

#### 3.4.2. Second: Incisor Retraction

##### Rate of the Incisor Retraction

One study compared the retraction rate of incisors between the two techniques [32]. The results showed that there was no significant difference between the retraction via the sliding technique on a 0.017 × 0.025-inch stainless steel wire and the retraction via a 0.017 × 0.025-inch TMA T-loops technique.

##### Anchorage Loss following Incisor Retraction

Two studies investigated anchorage loss during the retraction of incisors between the two techniques [27,32]. The results showed that there were no significant differences between sliding on 0.017 × 0.025-inch stainless steel wires or retraction using 0.017 × 0.025-inch TMA T-loops [27]. The results of the second study showed that the anchorage loss rate when retracting using 0.017 × 0.025-inch TMA T-loops was greater than sliding on 0.017 × 0.025-inch stainless steel archwire, with a mean of 0.5 mm per 0.88 mm of incisor retraction amount versus 0.1 mm/month per 0.68 mm of incisor retraction amount, respectively [32].

#### 3.4.3. Third: Retraction of the Upper Six Anterior Teeth

##### Rate of the En-Masse Retraction

Two studies investigated the rate of en-masse retraction. The first found that the retraction via the sliding technique on 0.019 × 0.025-inch stainless steel archwire was quicker than the frictionless technique by Connecticut New Archwire (CNA) mushroom loop archwire, with a mean of 0.74 mm/month compared to 0.39 mm/month [28]. The second study found no significant differences between the two techniques [31].

##### Anchorage Loss following En-Masse Retraction

Four studies investigated anchorage loss upon en-masse retraction [26,28,30,31]. The results mentioned that the retraction by the continuous T-loop fabricated with 0.017 × 0.025-inch TMA compared to the retraction with the sliding on 0.019 × 0.025-inch Stainless steel archwire caused a greater loss of anchorage (a mean of 2.44 ± 0.46 mm versus 0.95 ± 0.36 mm, respectively) [26], whereas in the second study, it was found that the retraction using the two-step technique (the canines sliding by power chain traction (100 g) then the incisors being closed with vertical loops and intermaxillary elastics) caused greater anchorage loss in both jaws compared to the en-masse retraction using the sliding technique on 0.016 × 0.022 inches of stainless steel [30]. When the sliding of the coil spring on 0.019 × 0.025 inches of stainless steel was compared with the CNA mushroom loop archwire, there were no significant differences [28,31].

##### Changes in Anterior Tooth Torque following En-Masse Retraction

Three studies investigated torque changes after en-masse retraction [28,30,31]. A significant reduction in the proclination of upper incisors was seen when retraction was performed using a 0.019 × 0.025-inch stainless steel wire compared to a CNA mushroom loop archwire (a mean of −12° versus −7°, respectively) [28]. Table 3 summarizes the results of the studies.

## 4. Discussion

To our knowledge, this is the first systematic review comparing the effectiveness of sliding versus frictionless methods in retracting the canines, incisors, or all six upper anterior teeth together.

### 4.1. Rate of Canine Retraction

The retraction of canines via the Gjessing spring had a higher rate of retraction than retraction via the sliding technique on a 0.018-inch wire within the 0.018 slot brackets. The reason may be attributed to the high frictional forces in the sliding technique generated from inadequate clearance due to the use of an archwire equal to the full height of the bracket slot [17]. Meanwhile, the retraction using the frictionless technique via the TMA T-loops spring had the lowest rate of retraction compared to the sliding on the 0.016 × 0.022-inch archwire [23]. This may be explained by the fact that when using the T-loops spring, the moment–force ratio was too high, causing a distal root movement of the canine greater than the crown movement, which was minimal [23]. The results were generally contradictory, not giving any conclusive evidence of the superiority of one of the two techniques. Given the value of the dental movement rate, which was very low (0.1 mm/month) for the T-spring in one of the two studies, it can be inferred that the retraction results using the sliding technique were good in the included studies, while the loop-based technique was a sensitive technique that may be rendered useless if poorly designed or fabricated.

### 4.2. Canine Tipping and Rotation Change

Ziegler et al. found that sliding on a 0.018-inch round section wire in 0.018-inch brackets caused higher tipping than retraction with a Gjessing spring [17]. This could be due to the sliding archwire’s low stiffness and the anti-tipping bend applied to the Gjessing spring. This bend has been applied to provide a force-to-moment ratio of 1:11, which provides biomechanically pure bodily movement [17]. Hayashi et al. found that there was no difference in the amount of distal tipping between the retraction with the sliding on a 0.018-inch archwire and with a Ricketts spring [29]. Both mean values of tipping change for both techniques in Hayashi’s study were higher than those in the first study (Ziegler’s study); this may be attributed to the slot height of the brackets in the sliding group in Hayashi’s study being 0.022 inches compared to 0.018 inches in Ziegler’s study. The difference between the bracket slot and the archwire used in Hayashi’s study led to high archwire play within the brackets, which may have caused a loss of control over bodily movement, thus resulting in a higher tipping motion. As for the relatively high tipping value obtained using the Ricketts spring compared to the Gjessing spring, this can be explained by the anti-tipping angle applied by the researcher in the Ricketts spring being 45° less than the ideal values of 90° recommended by Ricketts [33]. According to rotation control, canine rotation was significantly higher with a Ricketts spring than with sliding on the 0.018-inch stainless steel archwire [29]. The higher value of rotation may be explained by the anti-rotation value applied to the Ricketts spring being 45° lower than the ideal values. Therefore, it can be considered that to obtain good control of the canine tip movement, a high rigidity with a small field of play must be provided for the archwire in the sliding technique, and in the loop-based technique, the tips approved for the springs must be implemented.

### 4.3. Anchorage Loss of Canine Retraction

According to Ziegler’s study, there was no clinically significant anchorage loss for both techniques, which may be attributed to continuous anchorage reinforcement using the headgear [17]. Alhadlaq et al. found that the retraction by the frictionless technique caused significant anchorage control compared to the sliding technique [16]; the reason can be explained by the reinforcement of the anchorage that was performed using the beta bend in the T-loop spring, while the use of the TPA was mainly to control the rotation of the molars in both techniques. Therefore, limited evidence suggests that the loop-based technique can enhance the anchorage through the bends to achieve a geometry that secures the required anchorage model.

### 4.4. Root Resorption of Canine Retraction

The root resorption when using the frictionless technique with a TMA T-loop spring was similar to that when using the sliding technique [23]; the reason may be due to the use of force, continuous and light (150 g), within the recommended limits.

### 4.5. Anchorage Loss following Incisor Retraction

Bakhit et al. did not find a difference between retraction using sliding on 0.017 × 0.025-inch stainless steel wires or retraction using 0.017 × 0.025-inch TMA T-loops. This can be explained by the use of mini-screws, which provided absolute anchorage for the posterior segment [27]. In another similar study that used the same tools for retraction, a significant loss of anchorage was obtained when using the frictionless technique with 0.017 × 0.025-inch TMA T-loops [32]. This may be explained by direct loading to the first molars through the engagement of the beta arm of the T-loop, whereas in the sliding group, the loading was direct to the mini-screw for the retraction of the incisors. Therefore, if the treatment plan requires maximum anchorage during the retraction of the incisors, then mini-screws should be used.

### 4.6. Rate and Torque Change of En-Masse Retraction

Two studies investigated the en-masse retraction rate and concluded that a significant advantage in retraction speed was given when sliding on a flat 0.019 × 0.025-inch stainless steel archwire with a light force of 150 g [28]. However, this method had the least torque control compared to the frictionless technique using a CNA mushroom loop archwire [28]. The addition of a mild curve to the 0.019 × 0.025-inch stainless steel archwire led to its equivalence with the frictionless technique using a CNA mushroom loop in both the rate of retraction and the control of the torque [31].

### 4.7. Anchorage Loss following En-Masse Retraction

The anchorage loss was significantly less when using the sliding technique with 0.019 × 0.025-inch or 0.016 × 0.022-inch of stainless steel. [26,30]. This may be explained by the fact that the retraction forces in the sliding group were given directly from the posteriorly placed implants, while in the frictionless group, they were directly applied to the posterior segment.

### 4.8. Limitations

One notable limitation is that this review included only four randomized controlled trials out of the ten studies retrieved. Another limitation is that we were unable to perform a meta-analysis due to the different retraction methods used in the included studies. In addition, the total number of articles that investigated the retraction of incisors was very mall (i.e., two articles only), which did not help in building up a clear idea about the best retraction methodology.

## 5. Conclusions

Since the number of studies examining the differences between the various techniques is small, the evidence and information related to the superiority of one technique or method of retraction over another remain insufficient. In terms of orthodontic tooth movement rate, limited evidence pointed to the lasting effectiveness and overall superiority of the sliding technique and the high sensitivity of the design in the loop-based technique. According to the anchorage control, studies indicated that the sliding technique is similar to the loop-based technique in this aspect. The two techniques regarding canine tipping control had no clinically significant differences. As for the control of canine rotation, the evidence showed that the rotation of the canine was high when the specifications of the required anti-rotation bends were not followed. Regarding the control of the incisor’s torque during en-masse retraction, the available evidence is limited and indicates the superiority of the loop-based technique over the sliding one. Therefore, there is a need to conduct more randomized controlled trials with an appropriate parallel-group design, ensuring good randomization and the selection of patients with practically comparable retraction devices applied.

## Figures and Tables

**Table 1:** Keywords used in this search covering the important aspects regarding the population, outcomes, and interventions (under the PICOS framework).

Type of occlusion and malocclusion	Permanent occlusion, class II relationship, excessive overjet, maxillary dentoalveolar protrusion, bimaxillary protrusion, severe crowding, anterior open bite, first premolar extraction.
Treatment planning	Anterior teeth retraction, incisor retraction, canine retraction, en-masse retraction, moving anterior teeth backward, space closure.
Outcomes	Orthodontic tooth movement rate, orthodontic tooth movement amount, orthodontic tooth movement velocity, orthodontic tooth movement speed, orthodontic tooth movement duration, anchorage loss, rotation, inclination, torque, angulation, tipping, root resorption.
Interventions	Segmental technique, Segmented technique, sectional, Frictionless Mechanics, T-loop, L-loop, Loop-based technique, Ricketts’s spring, Gjessing retraction arch, Ladanyi spring, Marcotte spring, Reverse Closing Loop, Retraction spring.

**Table 2:** Characteristics of the included trials.

Authors	Number of Patients/Mean Age/Study Design	The Mechanism of Application/Force Intensity	Outcomes	Follow-Up Period	Extraction Time/Anchorage	Assessment Tool
Canine retraction studies						
Ziegler et al., 1989 [17]	21 (5 male, 16 female). Range: 10 to 27 y, median age: 13 y 6 m Split-mouth Class II malocclusion	Sliding M: on 0.018-inch (0.45 mm) S.S by PCT (Canines secured by ligatures to avoid rotations). Sectional A: Gjessing’s Retraction Spring Force magnitude: SM: 380 g → 200 g–SA: 160 g. The brackets were of the twin type with an 0.018-inch slot. The brackets used on the canines were 3.5 mm wide without angulation or torque. Sliding on 0.018 ss	Canine retraction rate, anchorage loss, tipping and rotation of the canines	Until completion of canine retraction	Immediately before retraction TPA + headgear was worn 10 to 14 h per day	Casts: T1: before movement; T2: after movement. Dental analysis was performed on photographed study models in a standard way. The angulation study was carried out clinically by the method of pin and bar.
Hayashi et al., 2004 [29]	8 (3 male, 5 female). Range: 19 y to 29 y Mean age: 23 y, 2 m Parallel-group: 2 groups Class II malocclusion	Sliding M: 0.018-inch S.S. Bracket: standard edgewise 0.022 × 0.028 Sectional A: Ricketts’s Canine Retraction Spring. Bracket: standard edgewise 0.018 × 0.025. Force magnitude: 1 N	Distal movement, tipping and rotation of canine	2 months	Osseo-integrated midpalatal implants	Impressions of the maxillary arch each week. A 3D surface-scanning system using a slit laser beam was used to measure the series of dental casts.
Alhadlaq et al., 2016 [16]	20 Parallel group: 2 groups (n = 10) Class II malocclusion	Sliding M: On 0.018 × 0.025-inch S.S. Sectional A: T-loop fabricated from 0.019 × 0.025-inch TMA (RMO) using synergy bracket system (0.022 × 0.025 in)	Ricketts cephalometric analysis (Anchorage loss by U6-PT Vertical)	Until completion of canine retraction	TPA	CRs were obtained at the beginning of the treatment (T0) and immediately after complete canine retraction (T1).
Makhlouf et al., 2018 [23]	10 (3 male, 7 female) Split-mouth Class II malocclusion	Sliding M: coil spring (on 0.016 × 0.022-inch S.S.) (left side) Sectional A: T-loops (0.017 × 0.025 TMA wires) (right side). Force magnitude: 150 g MBT 0.022	Amount of canine retraction, root resorption	(4 months).		CBCT pre-retraction and post-retraction
Incisor retraction studies						
Bakhit et al., 2022 [27]	40 (40 fe). Mean age: 15.6 y (friction group)/16 y (frictionless group) Bimaxillary protrusion RCT	Sliding M: PCT/on 0.017 × 0.025 S.S. Sectional A: 0.017 × 0.025-inch TMA T-loops. Force magn: SM-SA: 160 g. bracket system: Roth 0.022-inch	Anchorage loss, Torque changes of the incisors, ANB°, and B° change	After incisor retraction was completed and normal overjet obtained	Mini-screws	CBCT pre-retraction and post-complete retraction
Tawfik et al., 2022 [32]	30 (30 fe). Mean age: 18.3 ± 3.7 y. Parallel group: 2 groups (n = 15) Bimaxillary protrusion. RCT	Sliding M: PCT/on 0.017 × 0.025-inch S.S. Sectional A: 0.017 × 0.025-inch TMA T-loops. Force magn: 160 g. bracket system: Roth 0.022-inch	The rate of retraction, anchorage loss	Until the closure of the extraction space and establishment of normal overjet	TADs for indirect anchorage	Study models were scanned using a 3Shape R500 scanner.
En-masse retraction studies						
Koyama et al., 2011 [30]	28 (3 ma, 25 fe). Mean age: 24.9 ± 5 y. Parallel-group: 2 groups Bimaxillary protrusion En-masse retraction in group 1. Two-step retraction in group 2. Retrospective study.	Group 1 (Sliding M): PCT/on 0.016 × 0.022 inches of stainless steel. Bracket system: Edgewise 0.018-inch slot Group 2 (Sectional A): straight-pull headgear and intermaxillary elastics Two-step retraction (the canines by sliding by PCT (100 g) then the incisors were closed with vertical loops and intermaxillary elastics.	SNA, SNB, ANB, SN-MP°, SN-U1°, SN-L1°, U1 and U6 position, L1 and L6 position, overjet, and overbite changes	-	Sliding M: implant anchorage Sectional A: straight-pull headgear (200 g/12 h a day) and intermaxillary elastics	CRs analysis
Goyal et al., 2019 [28]	22 2 groups (n = 11) Class II malocclusion	Sliding M: coil spring/0.019 × 0.025 inches of stainless steel. Sectional A: CNA mushroom loop archwire. Force magn: 150 g	The rate of space closure. Anchorage loss. Torque changes	After 6 months of retraction	Indirect anchorage was taken from mini-screws	Lateral cephalograms were taken for each patient after 6 months of retraction. Study models and photographs
Arvind et al., 2021 [26]	40 2 groups (n = 20) Bimaxillary protrusion En-masse anterior retraction Retrospective study.	Sliding M: On 0.019 × 0.025 S.S. archwire with hooks. Sectional A: continuous T-loop fabricated with 17 × 25 TMA	Molar anchorage loss		Sectional A: TPA + inclusion of second molars to the anchor unit.	CRs analysis
Sardana et al., 2023 [31]	36 (10 ma, 26 fe). Parallel-group: 2 groups (n = 19) Angle’s Class II Division 1 and Class I bimaxillary dentoalveolar protrusion Malocclusion RCT	Sliding M: On 0.019 × 0.025-inch S.S. archwire with a mild curve of Spee. Sectional A: using 0.017 × 0.025-inch (CNA Beta Titanium) mushroom looped continuous archwire. bracket system: MBT 0.022-inch	The rate of en-masse retraction. Anchorage loss	The closure of the extraction spaces and normal overjet establishment	Maxillary second molars were bonded to augment anchorage, and a transpalatal arch was placed for transverse control	Study models and cephalometric

Y: years, m: month, ma: male, fe: female, Force magn: force magnitude, Sliding M: sliding mechanics, Sectional A: sectional archwire, CRs: cephalometric radiographs, CBCT: Cone-Beam Computed Tomography, PCT: Power Chain Traction.

**Table 3:** The main findings of the included trials.

Canine Retraction Studies				
Authors	Rate of Canine Retraction	Tipping *	Rotation	Anchorage Loss & Root Resorption
Ziegler et al., 1989 [17]	Sliding M: 1.4 mm/30 days Sectional A: 1.91 mm/30 days	Sliding M: 1.41 ± 1.29°/mm. Sectional A: 0.77 ± 0.82°/mm. Difference: 0.6°.	Sliding M.: 4.04 ± 2.37/mm. Sectional A.: 5.07 ± 1.50/mm. Nonsignificant	The average anchorage loss: Sliding M: 0.4 mm; Sectional A: 0.6 mm Anchorage loss per millimeter of canine retraction: Sliding M: 0.07 mm; Sectional A: 0.09 mm
Hayashi et al., 2004 [29]	Sliding M: 3.62 ± 0.19 mm/2 months Sect A: 3.95 ± 0.34 mm/2 months Nonsignificant	Sliding M: 7.94°/2 months. Sectional A: 7.89°/2 months. Nonsignificant	Sliding M: 4.07/2 months. Sectional A: 22.06/2 months. Significant	
Alhadlaq et al., 2016 [16]				Anchorage loss: Sliding M: 4.5 ± 3; Sectional A: −0.7 ± 1.4. In Sliding M, the upper first molars moved forward > Sectional A.
Makhlouf et al., 2018 [23]	Sliding M: 0.775 mm/month. Sectional A: 0.1 mm/month. Significant			Root resorption: Sliding M: 0.05 mm/month. Sectional A: 0.275 mm/month. Nonsignificant
Incisor retraction studies				
Authors	Rate of the retraction		Anchorage loss	
Bakhit et al., 2022 [27]			Sliding M: U6 MB = 0.593° L6 = 0.532° Sectional A: U6: 1.095° L6: 0.061° Nonsignificant	
Tawfik et al., 2022 [32]	Sliding M: 0.68 ± 0.18 mm/month. Sectional A: 0.88 ± 0.27 mm/month. Nonsignificant		Sliding M: 0.48 mm/4.8 month. Sectional A: 2.1 mm/4.3 month. significant	
En-masse retraction studies				
Authors	Rate of en-masse retraction	Torque changes	Anchorage loss	
Koyama et al., 2011 [30]		SN-U1°: Sliding M: −10.3 ± 5.8°; Sectional A: −11.1 ± 5.9°: nonsignificant SN-L1°: Sliding M: −6.8 ± 2.1°; Sectional A: −4.6 ± 3.0°: nonsignificant	Upper jaw: Sliding M: 0.1 ± 0.5 mm; Sectional A: 2.1 ± 1.3 mm: significant Lower jaw: Sliding M: 1.3 ± 1.3 mm; Sectional A: 2.5 ± 1.3 mm: significant	
Goyal et al., 2019 [28]	Sliding M: 0.74 mm/month; Sectional A: 0.39 mm/month: significant.	Sliding M: −12.73°; sectional A: −7.27°: significant.	Sliding M: 0.21 mm; Sectional A: 0.18 mm: nonsignificant	
Arvind et al., 2021 [26]			Sliding M: 0.95 ± 0.36 mm; Sectional A: 2.44 ± 0.46 mm: significant	
Sardana et al., 2023 [31]	Sliding M: 0.7 mm/month; Sectional A: 0.8 mm/month: nonsignificant	Sliding M: 10.9° ± 4.6°; Sectional A: 11.45° ± 3.9°: nonsignificant	Sliding M: 2.28 ± 238 mm; Sectional A: 1.13 ± 1.42 mm: nonsignificant	

*: The results express the amount of tipping change after retraction, as before during the mentioned observation period. Sliding M: Sliding Mechanics, Sectional A: Sectional Archwire.

## Data Availability

Datasets and spreadsheets underlying the current work are available upon reasonable request from the corresponding authors.

## Supplementary Materials

The following supporting materials can be downloaded at https://www.mdpi.com/article/10.3390/jcm12216757/s1, Table S1: The reasons behind the judgments regarding the risk of bias of the included randomized trials; Table S2: The reasons behind the judgments regarding the risk of bias of the included non-randomized trials.

## Appendix A

**Table A1:** Search strategy used in the current systematic review.

PubMed	#1 (permanent occlusion OR class II relationship OR excessive overjet OR maxillary dentoalveolar protrusion OR bimaxillary protrusion OR severe crowding OR anterior open bite OR first premolar extraction) #2 (Anterior teeth retraction OR incisors retraction OR canine retraction OR En masse retraction OR moving anterior teeth backward OR space closure) #3 (Orthodontic tooth movement rate OR orthodontic tooth movement amount OR orthodontic tooth movement velocity OR orthodontic tooth movement speed OR orthodontic tooth movement duration OR anchorage loss OR Rotation OR inclination OR torque OR angulation OR tipping OR root resorption) #4 (Segmental technique OR Segmented technique OR sectional OR Frictionless Mechanics OR T-loop OR L-loop OR Loop-based technique OR Ricketts’s spring OR Gjessing retraction arch OR Ladanyi spring OR Marcotte spring OR Reverse Closing Loop OR Retraction spring) #5 #1 AND #2 AND #3 AND #4
CENTRAL (The Cochrane Library)	#1 (permanent occlusion OR class Ⅱ relationship OR excessive overjet OR maxillary dentoalveolar protrusion OR bimaxillary protrusion OR severe crowding OR anterior open bite OR first premolar extraction) #2 (Anterior teeth retraction OR incisors retraction OR canine retraction OR En masse retraction OR moving anterior teeth backward OR space closure) #3 (Orthodontic tooth movement rate OR orthodontic tooth movement amount OR orthodontic tooth movement velocity OR orthodontic tooth movement speed OR orthodontic tooth movement duration OR anchorage loss OR Rotation OR inclination OR torque OR angulation OR tipping OR root resorption) #4 (Segmental technique OR Segmented technique OR sectional OR Frictionless Mechanics OR T-loop OR L-loop OR Loop-based technique OR Ricketts’s spring OR Gjessing retraction arch OR Ladanyi spring OR Marcotte spring OR Reverse Closing Loop OR Retraction spring) #5 #1 AND #2 AND #3 AND #4
Web of Science	#1TS= (permanent occlusion OR class Ⅱ relationship OR excessive overjet OR maxillary dentoalveolar protrusion OR bimaxillary protrusion OR severe crowding OR anterior open bite OR first premolar extraction) #2TS = (Anterior teeth retraction OR incisors retraction OR canine retraction OR En masse retraction OR moving anterior teeth backward OR space closure) #3TS = (Orthodontic tooth movement rate OR orthodontic tooth movement amount OR orthodontic tooth movement velocity OR orthodontic tooth movement speed OR orthodontic tooth movement duration OR anchorage loss OR Rotation OR inclination OR torque OR angulation OR tipping OR root resorption) #4TS = (Segmental technique OR Segmented technique OR sectional OR Frictionless Mechanics OR T-loop OR L-loop OR Loop-based technique OR Ricketts’s spring OR Gjessing retraction arch OR Ladanyi spring OR Marcotte spring OR Reverse Closing Loop OR Retraction spring) #5 #1 AND #2 AND #3 AND #4
Scopus	#1 TITLE ABS-KEY (permanent occlusion OR class II relationship OR excessive overjet OR maxillary dentoalveolar protrusion OR bimaxillary protrusion OR severe crowding OR anterior open bite OR first premolar extraction) #2 TITLE ABS-KEY (Anterior teeth retraction OR incisors retraction OR canine retraction OR En masse retraction OR moving anterior teeth backward OR space closure) #3 TITLE ABS-KEY (Orthodontic tooth movement rate OR orthodontic tooth movement amount OR orthodontic tooth movement velocity OR orthodontic tooth movement speed OR orthodontic tooth movement duration OR anchorage loss OR Rotation OR inclination OR torque OR angulation OR tipping OR root resorption) #4 TITLE ABS-KEY (Segmental technique OR Segmented technique OR sectional OR Frictionless Mechanics OR T-loop OR L-loop OR Loop-based technique OR Ricketts’s spring OR Gjessing retraction arch OR Ladanyi spring OR Marcotte spring OR Reverse Closing Loop OR Retraction spring) #5 #1 AND #2 AND #3 AND #4
EMBASE	#1 (permanent occlusion OR class II relationship OR excessive overjet OR maxillary dentoalveolar protrusion OR bimaxillary protrusion OR severe crowding OR anterior open bite OR first premolar extraction) #2 (Anterior teeth retraction OR incisors retraction OR canine retraction OR En masse retraction OR moving anterior teeth backward OR space closure) #3 (Orthodontic tooth movement rate OR orthodontic tooth movement amount OR orthodontic tooth movement velocity OR orthodontic tooth movement speed OR orthodontic tooth movement duration OR anchorage loss OR Rotation OR inclination OR torque OR angulation OR tipping OR root resorption) #4 (Segmental technique OR Segmented technique OR sectional OR Frictionless Mechanics OR T-loop OR L-loop OR Loop-based technique OR Ricketts’s spring OR Gjessing retraction arch OR Ladanyi spring OR Marcotte spring OR Reverse Closing Loop OR Retraction spring) #5 #1 AND #2 AND #3 AND #4
Google scholar	(permanent occlusion OR class II relationship OR excessive overjet OR maxillary dentoalveolar protrusion OR bimaxillary protrusion OR severe crowding OR anterior open bite OR first premolar extraction) AND (Anterior teeth retraction OR incisors retraction OR canine retraction OR En masse retraction OR moving anterior teeth backward OR space closure) AND (Orthodontic tooth movement rate OR orthodontic tooth movement amount OR orthodontic tooth movement velocity OR orthodontic tooth movement speed OR orthodontic tooth movement duration OR anchorage loss OR Rotation OR inclination OR torque OR angulation OR tipping OR root resorption) AND (Segmental technique OR Segmented technique OR sectional OR Frictionless Mechanics OR T-loop OR L-loop OR Loop-based technique OR Ricketts’s spring OR Gjessing retraction arch OR Ladanyi spring OR Marcotte spring OR Reverse Closing Loop OR Retraction spring)
Trip	(permanent occlusion OR class II relationship OR excessive overjet OR maxillary dentoalveolar protrusion OR bimaxillary protrusion OR severe crowding OR anterior open bite OR first premolar extraction) AND (Anterior teeth retraction OR incisors retraction OR canine retraction OR En masse retraction OR moving anterior teeth backward OR space closure) AND (Orthodontic tooth movement rate OR orthodontic tooth movement amount OR orthodontic tooth movement velocity OR orthodontic tooth movement speed OR orthodontic tooth movement duration OR anchorage loss OR Rotation OR inclination OR torque OR angulation OR tipping OR root resorption) AND (Segmental technique OR Segmented technique OR sectional OR Frictionless Mechanics OR T-loop OR L-loop OR Loop-based technique OR Ricketts’s spring OR Gjessing retraction arch OR Ladanyi spring OR Marcotte spring OR Reverse Closing Loop OR Retraction spring)
