# Supplementary material for: How Effective Are Non-Frictional Techniques Compared to Sliding Techniques in the Retraction of Upper Anterior Teeth When Using Buccal Fixed-Appliance Therapy? A Systematic Review
Source: J Clin Med. 2023 Oct 25;12(21):6757. doi: 10.3390/jcm12216757 (PMC10647228; doi:10.3390/jcm12216757)
Supplement: Supplementary file 1 [file jcm-12-06757-s001.zip › Supplementary Table S1.pdf]

Table S1: The reasons beyond the judges regarding the risk of bias of the included randomized trials

Study	Randomization process	Deviations from intended interventions	Missing outcome data	Measurement of the outcome	Selection of the reported result	Over-all bias
Ziegler and Ingervall 1989	Low risk: "The canine on one side, chosen at random, was retracted with a canine retraction spring and that on the other side along a labial arch…" There are no indications of a problem with the randomization process	Some concerns: The researchers did not report deviations that arose in the course of the experiment	Low risk: No dropouts were reported.	Some concerns: "The examiner is the one who applied the two therapeutic interventions and may have been influenced by this knowledge"	Some concerns: No information is available about the researchers' pre-specified intentions.	Some concerns
Bakhit et al. 2022	Low risk: " Randomization list was computer-generated using Microsoft Office Excel 2013 sheet. Allocation concealment was performed by co-author (HD) using opaque sealed envelopes…"	Low risk: We did not detect deviations from the intended intervention arising from the trial context.	Low risk: No dropouts were reported	Low risk: "… the assessors were blinded ..."	Low risk: The data were analyzed in accordance with a pre-specified plan that was finalized before unblinded outcome data were available for analysis	Low risk
Tawfik et al. 2022	Low risk: "This study was a two-arm, parallel, single-center, single-blind randomized clinical trial …"	Low risk: We did not detect deviations from the intended intervention arising from the trial context.	Low risk: No dropouts were reported	Low risk: " Two blinded external assessors carried out the measurements"	Low risk: "The study methodology was approved by the Faculty of Dentistry Ethical Committee, Future University in Egypt ([9]/10-2018). There were no	Low risk

					changes in methods after trial commencement."	
Sardana et al., 2023	Low risk:<br>" The randomization sequence was computer-generated (https://www.random.org/). Allocation concealment was done using opaque, sealed, and sequentially numbered envelopes. Allocation was handled by an affiliate not involved in any trial stage."	Low risk:<br>We did not detect deviations from the intended intervention arising from the trial context.	Low risk:<br>The number of patients completing the trial was consistent with the sample size needed for the study	Low risk:<br>"The outcome assessor who performed the measurements and the statistician were blinded to treatment allocation. The participants' information was anonymized using non-identifiable codes and removing identifying information at both levels."	Low risk"<br>The trial was registered at Clinical Trials Registry India, CTRI/2019/01/017231 and the outcomes mentioned in the protocol have been reported	Low risk
