# Supplementary material for: How Effective Are Non-Frictional Techniques Compared to Sliding Techniques in the Retraction of Upper Anterior Teeth When Using Buccal Fixed-Appliance Therapy? A Systematic Review
Source: J Clin Med. 2023 Oct 25;12(21):6757. doi: 10.3390/jcm12216757 (PMC10647228; doi:10.3390/jcm12216757)
Supplement: Supplementary file 1 [file jcm-12-06757-s001.zip › Supplementary Table S2.pdf]

Table S2: The reasons beyond the judges regarding the risk of bias of the included non-randomized trials

	Bias due to confounding	Bias in selection of participants into the study	Bias in classification of interventions	Bias due to deviations from intended interventions	Bias due to missing data	Bias in measurement of outcomes	Bias in selection of the reported result	Overall bias
Hayashi et al. 2004	Low risk: There are no obvious confounding factors that indicate a clear risk of bias.	Low risk: The selection of the participants was made before the start of the intervention. The initiation of follow-up and the start of the intervention coincided for the participants	Low risk: The intervention groups were clearly defined.	Low risk: Significant co-interventions were balanced across the intervention groups and the intervention was successfully implemented	Serious risk: The nature of the missing data means that the risk of bias cannot be removed through appropriate analysis.	Low risk: "A 3D surface-scanning system using a slit laser beam was used to measure the series of dental casts."	NI: Because the previously registered protocol cannot be accessed	Serious risk
Koyama et al. 2011	Low risk: There are no obvious confounding factors that indicate a clear risk of bias.	Moderate risk: Selection into the study may have been related to intervention and outcome and the authors used appropriate methods to adjust for the selection bias.	Moderate risk: Some aspects of the assignments of intervention status were determined retrospectively. .	Low risk: Any deviations from intended intervention reflected usual practice.	Low risk: Data were reasonably complete	Low risk: "The radiographs were traced by one person (RH), and 13 landmarks were identified and digitized with a protractor and digital caliper."	Low risk: the study is comparable to a well-performed randomized trial in this domain	Moderate risk
Alhadlaq et al. 2016	Low risk: There are no obvious	Low risk: The selection of the participants was	Low risk: The intervention	Low risk: Significant co-interventions	Low risk: The analysis addressed	Low risk: "All cephalometric	Low risk:	Low risk

	confounding factors that indicate a clear risk of bias	made before the start of the intervention. The initiation of follow-up and the start of the intervention coincided for the participants	groups were clearly defined.	were balanced across the intervention groups and the intervention was successfully implemented	missing data and is likely to have removed any risk of bias	radiographs were digitized using Dolphin imaging software and Ricketts cephalometric analysis was used"	"Every effort was made to minimize adjustments and to follow the treatment protocol strictly, at least during the canine retraction stage"	
Makhlouf et al. 2018	Low risk: There are no obvious confounding factors that indicate a clear risk of bias	Low risk: The sample for this study comprised of Ten adult patients in a split-mouth design (seven females, three males) with Class I or II division 1 malocclusion, and a treatment plan that necessitates the extraction of maxillary first premolars and retraction of permanent maxillary canines with moderate to minimum anchorage demand.	Low risk: Using a ligature wire, the T-loop was inserted and ligated into the right maxillary canine. On the right side, a closing coil spring 8 mm in length was attached to the first molar, and a force of 150 grams was used for retraction	Low risk: Significant co-interventions were balanced across the intervention groups and the intervention was successfully implemented	NI: There is no information about any missing data or exclusion of patients	Serious risk: The outcome measure was subjective And The outcome was assessed by assessors aware of the intervention received by study participants;	NI: Because the previously registered protocol cannot be accessed	Serious risk

Goyal et al., 2019	Low risk: There are no obvious confounding factors that indicate a clear risk of bias	Low risk: The selection of the participants was made before the start of the intervention. The initiation of follow-up and the start of the intervention coincided for the participants	Low risk: Intervention status is well-defined	Low risk: Significant co-interventions were balanced across the intervention groups and the intervention was successfully implemented	Low risk: Data were reasonably complete	Serious risk: The outcome measure was subjective And The outcome was assessed by assessors aware of the intervention received by study participants	NI: Because the previously registered protocol cannot be accessed	Serious risk
Arvind et al. 2023	Low risk: There are no obvious confounding factors that indicate a clear risk of bias	Moderate risk: Selection into the study may have been related to intervention and outcome and the authors used appropriate methods to adjust for the selection bias	Moderate risk: Some aspects of the assignments of intervention status were determined retrospectively.	Low risk: Any deviations from usual practice were unlikely to impact the outcome.	Low risk: Data were reasonably complete	Low risk: "The pretreatment and postretraction lateral cephalograms were traced and the discrepancies in landmark location and measurements were resolved by agreement with the principal guide."	Low risk: the study is comparable to a well-performed randomized trial in this domain	Moderate risk

NI: No Information
